# Drug-Induced Hidradenitis Suppurativa: A Case Report

**DOI:** 10.7759/cureus.49637

**Published:** 2023-11-29

**Authors:** Abraham Kisule, Vivek Kak, Chidamber Alamelumangapuram, Ciji Robinson

**Affiliations:** 1 Rheumatology, Henry Ford Health System, Jackson, USA; 2 Infectious Disease, Henry Ford Health System, Jackson, USA; 3 Internal Medicine, Henry Ford Health System, Jackson, USA

**Keywords:** crohn’s disease (cd), hidradenitis suppuritiva, hurley's staging, hurley stage, tumor necrosis factor-alpha (tnfα), african american, adalimumab

## Abstract

Hidradenitis suppurativa (HS) is a chronic, debilitating inflammatory disorder of the hair follicles that localizes to the intertriginous and anogenital regions of the body. Lesions are characterized by inflammatory nodules, subcutaneous abscesses, fibrosis, and sinus tracts. Crohn's disease (CD) is an idiopathic chronic inflammatory bowel disease that affects any part of the gastrointestinal tract. Multiple treatment options exist for CD, including monoclonal anti-tumor necrosis factor alpha (TNF-α) antibodies like adalimumab (Humira). Adalimumab is an anti-TNF agent that has been approved by the United States Food and Drug Administration (FDA) for the treatment of HS.

A 35-year-old African American male with a history of fistulizing CD presented to the hospital for evaluation of severe pain and purulent drainage from open sores in his bilateral axillary regions, groin, buttocks, and face for four days. He was on adalimumab for two years, during which time he noted the development of Hurley stage III HS. The physical exam was remarkable for a cachectic, painful-appearing male, with multiple abscesses on his lower jaw extending to his upper neck draining thick serosanguinous fluid, with similar findings in his bilateral axillary regions, bilateral groin, and perianal regions. He was treated with intravenous antibiotics consisting of a fourth-generation cephalosporin and vancomycin. While the etiology of HS in this patient is inconclusive, the timing of its development closely aligns with the initiation of Humira and is not a manifestation of CD. Paradoxical adverse effects describe a phenomenon in which a medication can induce a condition that it classically can be used to treat. In this patient's case, it was HS.

## Introduction

Hidradenitis suppurativa (HS) is a chronic, debilitating inflammatory disorder of the hair follicles that localizes to the intertriginous and anogenital regions of the body. Lesions are characterized by inflammatory nodules, subcutaneous abscesses, fibrosis, and sinus tracts. Crohn's disease (CD) is an idiopathic chronic inflammatory bowel disease that affects any part of the gastrointestinal tract. Multiple treatment options exist for CD, including monoclonal anti-tumor necrosis factor-alpha (TNF-α) antibodies like adalimumab (Humira). Adalimumab is approved by the United States Food and Drug Administration (FDA) for the treatment of HS in patients aged 12 and older with moderate to severe HS [1].

Humira specifically targets and blocks TNF-α, which is a pro-inflammatory cytokine. By blocking TNF-α, Humira reduces inflammation, which is thought to contribute to HS symptoms and help manage HS [2].

## Case presentation

**Figure 1:**
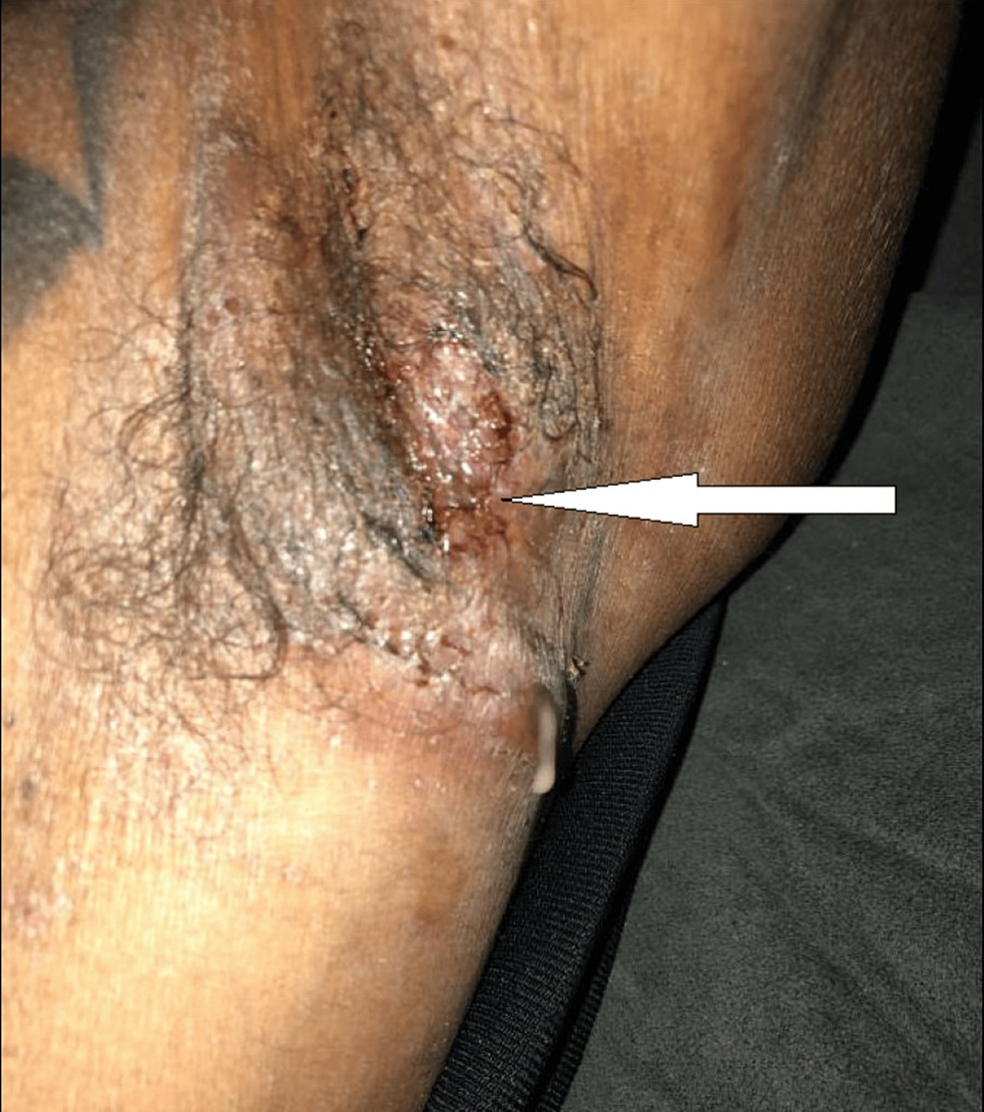
HS lesion in the left axillary region Arrow pointing to HS lesion with purulent drainage in the left axillary region. HS: hidradenitis suppurativa

**Figure 2:**
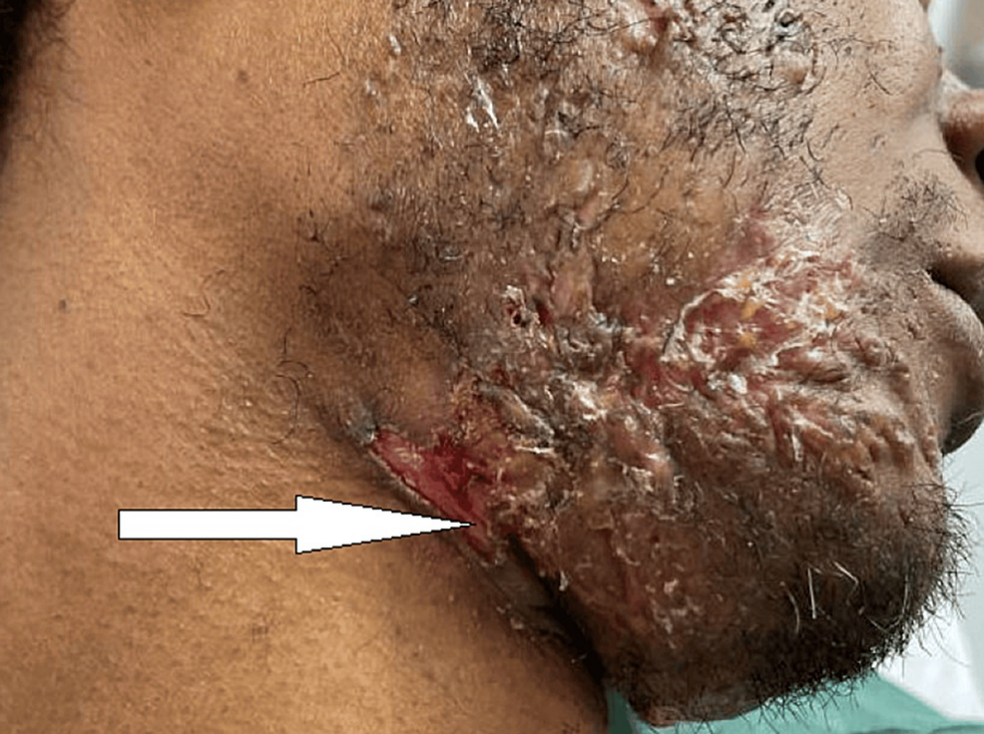
HS lesions on the neck region Arrow pointing to HS lesion on the submental/neck region HS: hidradenitis suppurativa

A 35-year-old African American male with a medical history of fistulizing Crohn's disease presented to the hospital for evaluation of severe pain and purulent drainage from open sores in his bilateral axillary regions (Figure 1), groin, buttocks, and face (Figure 2) for four days.

**Figure 3:**
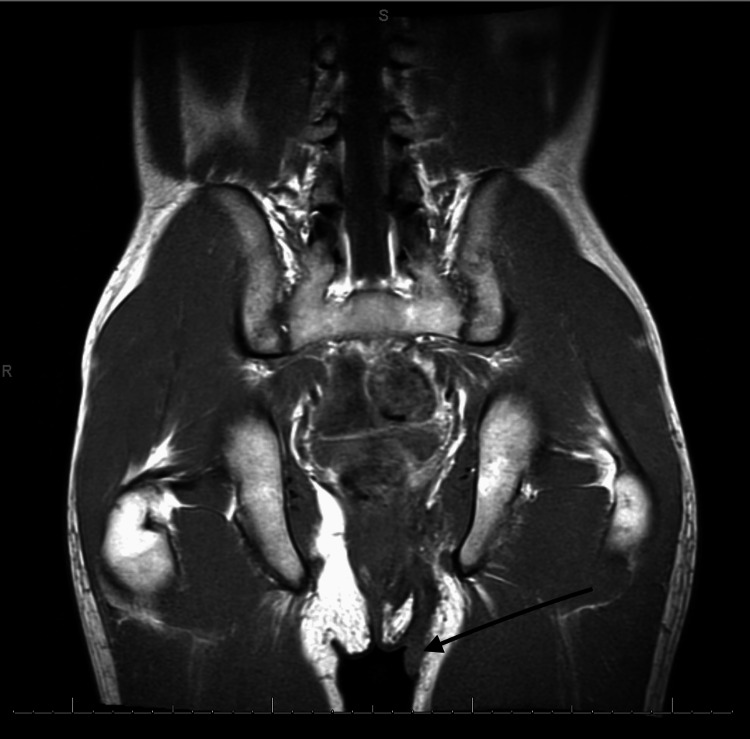
MRI of the pelvis shows a fistulous tract extending from the left pubococcygeal muscle into the left upper thigh, suggesting an abscess (arrow).

His Crohn's disease was initially treated with azathioprine but did not achieve adequate disease remission. He was then switched to adalimumab, on which his disease was better controlled for two years. In those two years, he noted the development of acneiform lesions on his face. The alleged acne was treated with isotretinoin and antibiotics and had disease progression despite adequate treatment. His gastroenterologist then referred him to a dermatologist for further evaluation of his uncontrolled acne. A biopsy was taken of the wounds, which turned out to be Hurley stage III HS. A physical exam was remarkable for a cachectic male in acute distress, with multiple abscesses on his lower jaw extending to his upper neck, draining thick serosanguinous fluid, and similar findings in his bilateral axillary regions, bilateral groin, and perianal regions. An MRI of the abdomen and pelvis showed a fistulous tract extending from the left pubococcygeal muscle into the left upper thigh, suggesting an abscess (Figure 3).

He was treated with intravenous antibiotics consisting of a fourth-generation cephalosporin and vancomycin. Wound cultures from the abscesses grew coagulase-negative *Staphylococcus *and Group B *Streptococcus sp*.. A punch biopsy obtained from a prior hospitalization of his axillary skin showed acute and chronic cellulitis compatible with HS. At discharge, we confirmed that the Humira had been discontinued. Additionally, he was asked to see a colorectal surgeon for a diverting colostomy to address the fistulous track in the left pubococcygeal muscle.

## Discussion

While the etiology of HS in this patient is inconclusive, based on the timing of the presentation of HS, there is a stronger correlation of HS lesions to the use of Humira versus a manifestation of CD. The fistulous tract etiology was more likely due to CD. Furthermore, the patient was diagnosed with CD in 2001 and had no HS until 2020, after he started therapy with Humira.

According to a literature review, Humira use has caused two cases of HS. One was a 57-year-old African American female with a history of CD who was started on mesalamine suppositories and adalimumab to treat her CD. She had disease remission on adalimumab, but unfortunately, 12 months into her treatment, she developed recurrent boils and nodules in her groin. She was then referred to dermatology, and the diagnosis of HS was confirmed by biopsy. She was placed on multiple antibiotics, with minimal improvement in her lesions. Thirty months later, erythematous lesions had manifested on her back at the site of the injection. Another case reported by the same authors involved a 24-year-old African American male with CD who was initially treated with 2.4 g of mesalamine daily. While on this regimen, he experienced disease progression manifested by abdominal pain, diarrhea, and the development of an anal fistula. His therapy was then changed to adalimumab for the treatment of moderate-to-severe CD. Nine months into treatment with adalimumab, he developed multiple sinus-draining nodules in his bilateral groins consistent with HS [3].

Paradoxical adverse effects describe a phenomenon in which a medication can induce a condition that it classically can be used to treat [4]. It was HS, in this patient's case. Although the mechanism behind this phenomenon is not fully understood, it is proposed to be secondary to the modulation of the immune system, resulting in an imbalance in cytokine levels and leading to a pro-inflammatory state [4].

There have been several studies implicating human leukocyte alleles (HLAs) and their association with various medications as the center of adverse drug reactions (ADRs). Most of the HLA-associated ADRs have ethnic specificity [5]. While there is a small sample size to draw such a conclusion in the cases mentioned in this article, it is difficult to rule that out as the etiology underlying the onset of HS in the 35-year-old patient.

We have seen pharmacogenomics being used in psychiatry, where patients undergo genetic testing to figure out to which antidepressants they would respond best. The Journal of the American Medical Association (JAMA) highlighted the results of a research study titled Effect of Pharmacogenomic Testing for Drug-Gene Interactions on Medication Selection and Remission of Symptoms in Major Depressive Disorder [6]. This randomized clinical study included 1,944 participants with major depressive disorder (MDD). Results of the study showed that the provision of pharmacogenomic tests for drug interactions compared with usual care resulted in prescriptions with no predicted drug-gene interactions compared with usual care in 45% vs. 18%, respectively.

## Conclusions

One can argue on behalf of the need to have patients undergo HLA genotype testing before consideration is given to starting them on a given monoclonal antibody medication. Pharmacogenomic testing will come at an added cost to an already expensive healthcare system. This price is nowhere near the one associated with plastic surgery to repair a disfigured part of the body. Adverse reactions, like those of the patient highlighted in this case report, can be disabling.
